# Factors Affecting Patients’ Selection of an Ophthalmologist in Saudi Arabia: A Patient-Centered Cross-Sectional Study

**DOI:** 10.7759/cureus.109108

**Published:** 2026-05-18

**Authors:** Yazeed A AlFerayan, Meshari A Alharbi, Abdulhamid H Altowairqi, Wael S Alharbi, Saleh A AlKhaldi

**Affiliations:** 1 Ophthalmology, King Fahad Medical City, Riyadh, SAU; 2 College of Medicine, Qassim University, Buraydah, SAU; 3 Medicine, Taif University, Taif, SAU; 4 Ophthalmology, Northern Border University, Riyadh, SAU; 5 Research Center, King Fahad Medical City, Riyadh, SAU

**Keywords:** communication, healthcare decision-making, ophthalmologist selection, patient preferences, saudi arabia

## Abstract

Background: Patients' preferences play a major role in selecting health care providers, yet, to our knowledge, no prior research in Saudi Arabia has examined which factors most influence the choice of an ophthalmologist. This study aimed to identify the key determinants guiding patients' decisions and explore demographic differences.

Methods: An analytical cross-sectional survey was conducted from September 2025 to February 2026, among adults who had visited an ophthalmology clinic in the past 12 months. A total of 418 participants completed an online questionnaire assessing sociodemographic data, clinical background, Likert-scale ratings of 28 selection factors, and the top three most influential factors. Fisher's exact test and the Mann-Whitney U test were used, with significance set at p<0.05.

Results: Communication quality and clinical competence were the strongest influences. The most important factors were taking enough time to answer questions (90.7%), availability of modern equipment (88.3%), clinic cleanliness (88.0%), and clear explanation of test results (87.1%). Clinical expertise indicators, including years of experience (84.0%), subspecialty fellowship (78.7%), and low complication rates (76.1%), were also highly valued. The top three selected factors were years of experience (56.2%), subspecialty fellowship (53.6%), and board certification (40.4%). Social media presence and cost-related factors had a limited influence.

Conclusion: Patients in Saudi Arabia prioritize professional expertise, effective communication, and a clean, well-equipped clinical environment when choosing an ophthalmologist. Convenience, cost, and social media play a secondary role. Strengthening clinical credibility and patient-centered communication may enhance patient trust and decision-making in ophthalmic care.

## Introduction

Patient motivation to consult with physicians is a critical component in disease management and prevention. A patient’s sense of comfort and confidence in their physician significantly contributes to the overall quality of healthcare. From this perspective, selecting an appropriate doctor may lead to improved treatment outcomes, greater satisfaction, and better adherence to treatment plans [1-3].

One of the most important consequences of allowing patients to choose their treating physicians is that it can increase competition among healthcare providers, which may, in turn, lead to higher service quality and patient satisfaction. Multiple studies have demonstrated that several factors influence a patient’s choice of healthcare provider [4-7]. Notably, these preferences are often shaped independently of the patient's current health condition, as people may select physicians before becoming ill [8].

It has been reported that when patients choose their health care plan, their overall satisfaction increases. Yet, when physicians choose the health care plan for patients, the overall satisfaction is negatively affected [9]. Since healthcare systems vary across countries, the influencing factors may also differ accordingly. Additionally, limited access to information about doctors may restrict patients' options and complicate the decision-making process.

According to a study by Bornstein et al. from Louisiana State University, several factors influence physician choice. The most highly rated variables were board certification, the physical attributes of the doctor's office, and the doctor's appearance [3]. On the other hand, another study by Mercado et al. reported that good communication and patient care are equally essential to all patients. Practices need to emphasize these features and patient education [4].

In a 2010 study, one-third of participants believed that a doctor's knowledge and experience are the most crucial factors. Other significant factors included giving patients enough time for the examination and showing a special interest in their problems [5].

In a survey including 10,205 patients, participants who chose their physicians (46.5%) rated their satisfaction as excellent or very good, whereas the satisfaction rate for those who were assigned a physician was good (54.5%). This was related to the personal choice of patients, rather than reasons such as physicians with higher patient satisfaction being chosen more often, health beliefs, demographic or socioeconomic differences, or health values [9].

One study conducted in Saudi Arabia that included 1582 participants stated that personal or family experience with the physician (reported by 86% of respondents) was the most influential factor, followed by the physician's title and area of specialization (82.4%). These elements played a substantial role in how patients chose their treating physicians [6]. Another Saudi study examining social media users found that a substantial proportion considered ophthalmologists' social media pages very important or extremely important when choosing an ophthalmologist [7].

In Saudi Arabia, patients in the private health sector tend to choose their physician and decide which health center to visit, since they do not have an assigned physician. To the best of our knowledge, no previous studies have investigated factors that may affect patients in choosing their treating physicians in the ophthalmology field. Therefore, the aim of this study was to explore the most influential factors that affect patients' decisions when selecting an ophthalmologist and to examine possible associations between the studied factors and demographic characteristics.

## Materials and methods

**Figure 1:**
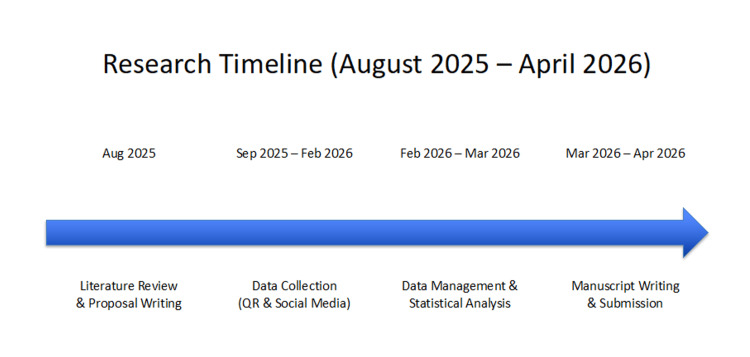
Research timeline

An analytical cross-sectional multicenter survey was conducted in several hospitals, such as King Fahad Medical City (Riyadh), Prince Mohammed bin Abdulaziz Hospital (Riyadh), Eye Specialist Hospital (Jeddah), Central Hospital (Buraydah), and Prince Abdulaziz bin Musaed Hospital (Arar), in Saudi Arabia. The data were collected between September 2025 and February 2026 (Figure 1). The aim was to evaluate the relative significance of factors influencing Saudi Arabian patients' selection of an ophthalmologist.

The questionnaire used in this study is verified and validated, and adapted from previously published studies [10]. It included 21 items divided between five dimensions (see the Appendices). The dimensions pertained to participants’ sociodemographic characteristics, decision not to seek medical treatment, and choice of healthcare providers. Questionnaire items were extracted from 'An Analysis Report of National Health Services Survey in China, 2008 (NHSS)', which was published by the National Health and Family Planning Commission in China, and revised according to the findings of a preliminary investigation. Various basic characteristics, including sex, occupation, age, monthly income, marital status, and educational level, were assessed to examine participants’ sociodemographic characteristics, which were completely consistent with the NHSS content [10,11].

The data collection tool was disseminated through online platforms to the patients of ophthalmology clinics across several hospitals in Saudi Arabia. In addition, they were approached in waiting areas and given a verbal explanation about the nature of the study. All subjects signed the consent form if they agreed to take part, and they had the right to withdraw at any point without any consequences.

The inclusion criteria were age ≥18 years and the ability to provide informed consent and complete the survey. Ophthalmologists/ophthalmic staff or trainees and patients with cognitive impairment or severe visual/communication limitations, preventing valid survey completion, were excluded.

Statistical considerations

A minimum sample size of 384 was calculated, using the following formula and a 95% confidence level:

$n = \frac{Z^2p(1-p)}{d^2}$.

Here, n is the calculated sample size; Z is the Z-value for the selected level of confidence (1 - a) = 1.96; p is estimated knowledge, Q is (1 - 0.50) = 50%, i.e., 0.50; d is the maximum acceptable error = 0.05:

$n = \frac{(1.96)^2 \times 0.50 \times 0.50}{(0.05)^2} = 384$.

Data were analyzed using IBM SPSS Statistics, version 25 (IBM Corp., Armonk, USA). The normality of data was assessed using the Shapiro-Wilk and Kolmogorov-Smirnov tests, which indicated non-normal distribution. Categorical variables were summarized as frequencies/percentages, sex differences were tested using Fisher's exact test, and age differences were evaluated using the Mann-Whitney U test. A p-value of less than 0.05 was considered statistically significant.

Ethical approval

Ethical approval (#25-484) was obtained from the IRB at King Fahad Medical City, where the study was coordinated. Informed consent was obtained from all participants before the start of the study. Data were securely saved in accordance with ICH (International Council for Harmonisation of Technical Requirements for Pharmaceuticals for Human Use) guidelines and Good Clinical Practice requirements. In addition, the confidentiality of the data was assured, and the privacy of the subjects was protected.

## Results

The study recruited 418 participants, with 48.6% male and 51.4% female participants. The mean age was 32.74 ± 11.68 years. Most participants had a bachelor's degree (72.0%), followed by secondary education (14.1%) and postgraduate studies (7.2%). Regarding occupation, doctors (23.9%), students/interns (23.2%), teachers (20.6%), and other occupations (13.1%) were the largest groups. The highest proportion of respondents (43.3%) reported a monthly income of 10,000-20,000 SAR, whereas 25.8% reported less than 3000 SAR. Most respondents did not have health insurance (78.2%) and had not undergone prior eye surgery (83.5%). In addition, 89.7% had previously visited an eye doctor, indicating prior experience with ophthalmic services.

The causes of the present visit were rather equally distributed as routine eye examinations (37.6%), and visits to medical treatment (36.8%) were a bit more frequent than surgical visits (25.6%). Nearly half of the respondents (46.4%) were visiting an ophthalmologist for the first time, which implies that many of the participants were already in a patient-provider relationship. On the whole, these results indicate that the study population consisted mostly of young and educated adults who had experience of using eye care services before (Table 1).

**Table 1:** Sociodemographic and clinical characteristics of participants (N=418)

Characteristic	n (%)
Age (years), mean ± SD	32.74 ± 11.68
Age (years), median (IQR)	28.00 (24.00–40.00)
Female sex	215 (51.4)
Male sex	203 (48.6)
Bachelor’s degree	301 (72.0)
Secondary education	59 (14.1)
Postgraduate studies	30 (7.2)
Diploma	24 (5.7)
Primary education	4 (1.0)
Doctor	100 (23.9)
Teacher	86 (20.6)
Student	61 (14.6)
Medical student/intern	36 (8.6)
Engineer	19 (4.5)
Housewife	13 (3.1)
Administrative work	13 (3.1)
Nurse	12 (2.9)
Retired	10 (2.4)
Military	7 (1.7)
Other	42 (10.0)
Not working	19 (4.5)
Monthly income, <3000 SAR	108 (25.8)
Monthly income, 3000–9999 SAR	84 (20.1)
Monthly income, 10,000–20,000 SAR	181 (43.3)
Monthly income, >20,000 SAR	45 (10.8)
No health insurance	327 (78.2)
Prior eye surgery	69 (16.5)
Ever visited an eye doctor	375 (89.7)
Eye-surgery consultation	96 (25.6)
Medical treatment visit	138 (36.8)
Routine eye examination	141 (37.6)
First visit to the current ophthalmologist	194 (46.4)

Importance of factors when choosing an ophthalmologist

When responses rated as very important or extremely important were combined, professional competence and interpersonal aspects emerged as the most influential factors in choosing an ophthalmologist. The highest levels of importance were observed for taking sufficient time to answer patients' questions (90.7%), availability of modern equipment (88.3%), clinic cleanliness and environment (88.0%), and explaining test results in simple language (87.1%). Clinical expertise indicators were also highly valued, including years of professional experience (84.0%), subspecialty fellowship training (78.7%), use of advanced surgical techniques (77.0%), low complication rates (76.1%), and board certification in ophthalmology (70.1%). Service-related and institutional factors such as center reputation (75.6%), recommendations from family, friends, or other doctors (76.1%), shorter waiting time for appointments (71.1%), and quality of reception or call-center services (61.7%) also influenced decision-making.

Financial and access-related factors had a moderate impact, including acceptance of insurance or reasonable prices (58.9%), out-of-pocket service cost (58.6%), convenient clinic location (59.8%), evening or weekend appointment availability (63.6%), and promotional offers (52.2%). In contrast, online presence had less influence: social media presence (33.7%), website or professional educational content (37.6%), Google reviews (45.9%), and social media recommendations (24%-27%) were less frequently rated as very important or extremely important (Table 2).

Overall, the findings indicate that perceived clinical expertise, quality of communication, and the care environment are the primary determinants of ophthalmologist choice, whereas social media influence and marketing factors play a comparatively limited role (Table 2).

**Table 2:** Factors rated as very important or extremely important when choosing an ophthalmologist OCT: optical coherence tomography

Factor	Very/extremely important, n (%)
Takes enough time to answer my questions	379 (90.7)
Availability of modern equipment	369 (88.3)
Clinic/hospital cleanliness and environment	368 (88.0)
Explains test results in understandable language	364 (87.1)
Years of professional experience	351 (84.0)
Subspecialty fellowship	329 (78.7)
Uses advanced surgical techniques	322 (77.0)
Low complication rate in operations	318 (76.1)
Recommendations from family/friends or other doctors	318 (76.1)
Uses modern diagnostic equipment (e.g., OCT)	314 (75.1)
A relative’s or friend’s experience with this center	311 (74.4)
Center’s reputation in the community	316 (75.6)
Short waiting time for appointments	297 (71.1)
Board certification in ophthalmology	293 (70.1)
Availability of evening or weekend appointments	266 (63.6)
Quality of reception service or call center	258 (61.7)
Convenient clinic location	250 (59.8)
Accepts insurance/reasonable prices	246 (58.9)
Cost of service not covered by insurance	245 (58.6)
Offers and discounts	218 (52.2)
Affiliation with a government or private hospital	210 (50.2)
Google ratings and reviews	192 (45.9)
Previous research or scientific publications	180 (43.1)
Website or professional educational content	157 (37.6)
Presence on social media	141 (33.7)
Recommendations via X	111 (26.6)
Recommendations via TikTok	107 (25.6)
Recommendations via Snapchat	102 (24.4)

Most influential factors affecting patients’ choice of an ophthalmologist

**Figure 2:**
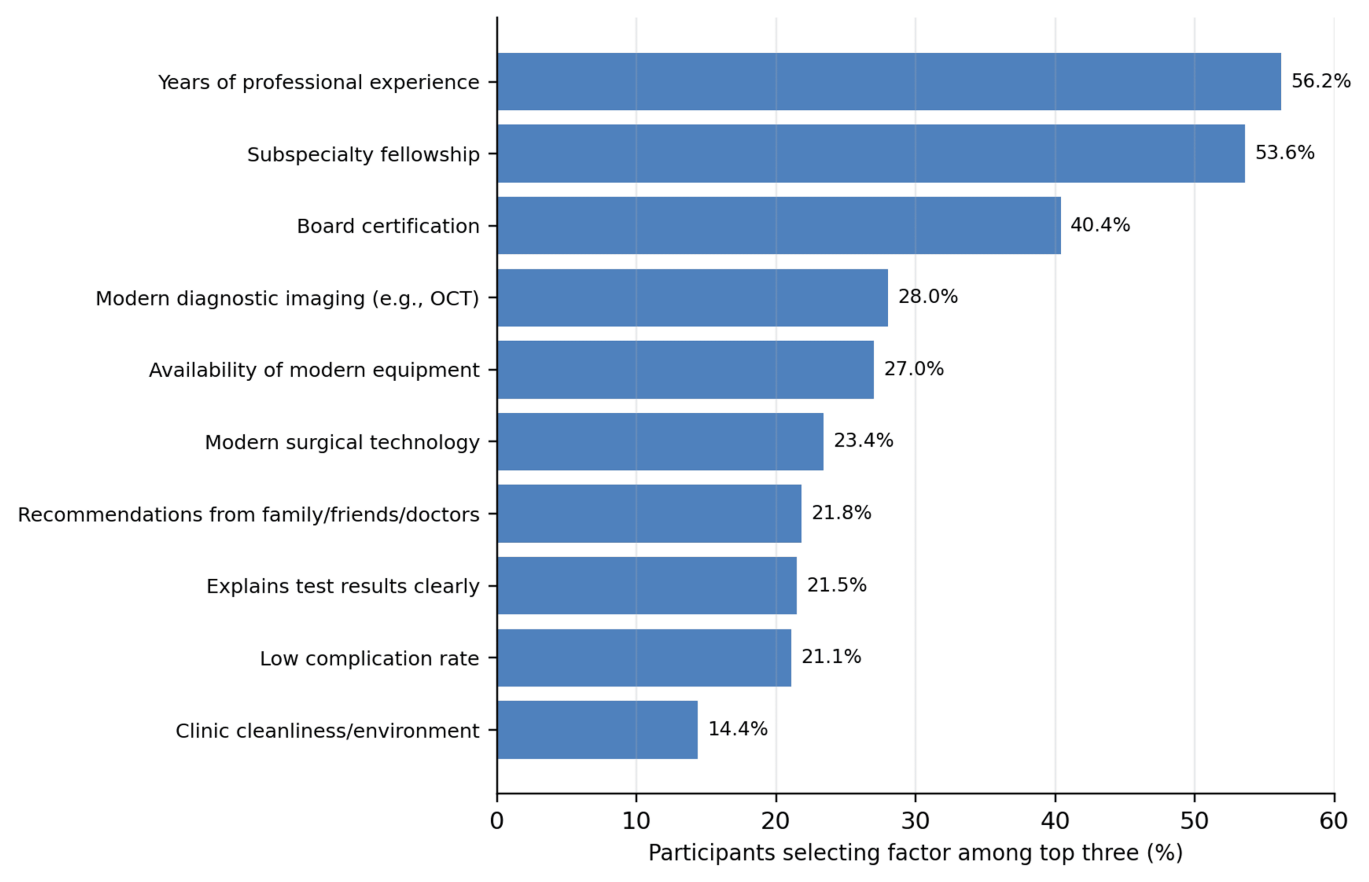
Top 10 most influential factors considered by participants when choosing an ophthalmologist (based on top-three selections) Horizontal bars show the proportion of participants who selected each factor among their top three most influential determinants. Image credits: Created by the authors using a non-generative workflow in Python (matplotlib).

When participants were asked to select up to three factors that most influenced their choice of an ophthalmologist, professional qualifications and clinical expertise clearly dominated decision-making. Years of professional experience was the most frequently selected factor (56.2%), followed by subspecialty fellowship training (53.6%) and board certification in ophthalmology (40.4%). Modern diagnostic imaging, such as optical coherence tomography (OCT; 28.0%), availability of modern equipment (27.0%), and use of modern surgical technology, such as femto-LASIK for vision correction and phacoemulsification (Phaco) or femtosecond laser-assisted cataract surgery (FLACS) for cataract extraction (23.4%), were also commonly selected. Interpersonal and trust-related factors, including recommendations from family, friends, or other doctors (21.8%), clear explanation of test results (21.5%), and low complication rates (21.1%), were less frequently chosen than formal expertise markers. These findings reinforce that perceived clinical competence and specialized expertise are the dominant drivers of patient choice (Figure 2).

Interestingly, the analysis of responses based on sex revealed that many factors differed significantly between female and male participants including affiliation with government (57.2% vs. 42.9%; p=0.004), quality of reception service (67.9% vs. 55.2%; p=0.009), convenient clinic location (65.6% vs. 53.7%; p=0.017), and Google rating and reviews (50.7% vs. 40.9%; p=0.045).

## Discussion

This study investigated what patients prioritize when selecting an ophthalmologist. Priorities such as communication quality, time investment, and care environment are core components of perceived service quality and satisfaction, as patients reported, often acting through trust as a mediator [12-16].

Perceived clinical risk is amplified by the stakes of vision, frequent use of diagnostic tests, and procedure-oriented care pathways. Patients, therefore, rely on competence cues they can personally verify [17]. Communication clarity, opportunities to ask questions, and adequate consultation time reflect diagnostic confidence and willingness to share decision responsibility. This mechanism helps explain why our participants rated time-to-answer questions and simple explanations at the top of all factors. Similar themes have been reported previously in the literature [18,19].

A second mechanism is cognitive load management. Eye care commonly involves unfamiliar, complex terminology, including imaging modalities that are available in the market, such as OCT, corneal topography, and intraocular lenses (i.e., monofocal, bifocal, or multifocal). Clear explanations reduce perceived complexity, strengthen comprehension, and support adherence to follow-up and treatment. This interpretation is consistent with recent work linking patient-centered communication to satisfaction and trust outcomes [12,13,16].

Availability of modern equipment and surgical techniques ranked high in our cohort. These items are classic structural quality cues, as patients can see equipment, associate it with up-to-date care, and infer better outcomes even if they cannot directly evaluate technical competence. Studies on ophthalmology clinic satisfaction similarly highlight the salience of facility readiness and perceived service environment [18,19].

Clinic condition and safety were another top-rated factor. Cleanliness likely operates as a safety heuristic, especially in procedure-heavy clinics where patients anticipate injections, minor procedures, or surgical planning [20]. Evidence from health services research indicates that cleanliness has a measurable association with satisfaction and can influence willingness to return, and recent modelling work suggests cleanliness effects may operate through trust [14,15].

Female participants placed more weight on institutional affiliation, reception or call center quality, clinic location, and Google reviews. These differences may reflect a higher sensitivity to system-level reliability and the full-service journey, including access logistics and front desk interactions. As indicated by the participants of our study, the top three selections for them were years of professional experience, subspecialty fellowship, and board certification. Patients cannot observe diagnostic reasoning directly, so they lean on verifiable credentials that are culturally understood as competence markers. This pattern is consistent with the previous literature [21,22].

Our results show that reputation and recommendations from family, friends, or other physicians were meaningful, but largely secondary to direct care experience signals. This is unsurprising as word of mouth reduces search costs and partially resolves uncertainty, but the information is still filtered through the patient’s need to confirm competence at the visit itself [23]. Ophthalmology review ecosystem studies support this hierarchy, showing that patient narratives often emphasize communication, perceived empathy, and clinic flow as key drivers of evaluations [23-25].

Despite global growth in social media health content, our cohort rated online presence as less important than interpersonal and structural quality cues. Several mechanisms may explain this. First, credibility concerns and variable content quality reduce the diagnostic value of social media signals. Second, social media may influence earlier awareness but not final selection when higher trust sources such as credentials, facility cues, and interpersonal experience become available [26-28].

Study limitations

This study is not without limitations. Reported preferences may not perfectly predict real-world choice behavior. Self-reported Likert ratings are vulnerable to social desirability and acquiescence bias. Convenience sampling and a sample composition skewed toward younger, highly educated respondents, and a high uninsured proportion, which may limit generalization.

Future directions

Future work can strengthen inference by using discrete choice experiments or conjoint designs to quantify trade-offs between credentials, access, cost, and experience attributes rather than testing each factor in isolation. Multivariable modelling can evaluate independent predictors of high importance ratings while adjusting for confounding and interaction, especially for age and gender effects. Longitudinal designs could link stated preferences to downstream outcomes such as adherence, follow-up completion, switching behavior, and patient-reported experience measures.

Older participants in our cohort were more likely to prioritize professional experience, modern diagnostic equipment, community reputation, and short waiting time. This is consistent with risk calibration and burden of disease logic, as older patients have a higher baseline prevalence of chronic eye disease and may anticipate more complex care episodes, making technology, experience, and operational efficiency more valuable [29]. The wait time effect is strongly supported in eye care settings, including quality improvement work and ratings analyses linking shorter waits to higher satisfaction and better public evaluations [30].

## Conclusions

Participants selected ophthalmologists primarily through signals that reduce uncertainty and build trust. Adequate time for questions, clear explanation of results, modern equipment, clinical environment, credentials, and subspecialty training were effective, while convenience and cost mattered moderately. Targeted improvements in communication, clinic operations, and transparent credential signalling are the most direct levers to align ophthalmology services with patient priorities.

## Appendices

A survey of outpatients’ healthcare‑seeking preferences

Section 1: Demographic Information

Age: ☐ <18 ☐ 18-29 ☐ 30-39 ☐ 40-49 ☐ 50-59 ☐ ≥60

Gender: ☐ Male ☐ Female

Education level: ☐ Primary school or below ☐ Middle school ☐ High school ☐ College diploma ☐ Bachelor’s degree ☐ Postgraduate degree

Employment status: ☐ Employed ☐ Self‑employed ☐ Student ☐ Retired ☐ Unemployed

Monthly household income: ☐ <3000 RMB ☐ 3000-4999 RMB ☐ 5000-7999 RMB ☐ 8000-11999 RMB ☐ ≥12000 RMB

Health insurance type: ☐ Urban employee insurance ☐ Urban resident insurance ☐ New rural cooperative insurance ☐ Commercial insurance ☐ None

Section 2: Healthcare‑Seeking Behavior

When you feel unwell, which type of healthcare facility do you usually visit first? ☐ Community health center ☐ Secondary hospital ☐ Tertiary hospital ☐ Private clinic ☐ Other: _________

Main reason for choosing this type of facility: ☐ Better medical technology ☐ More experienced doctors ☐ Shorter distance ☐ Lower cost ☐ Covered by insurance ☐ Habit / previous experience ☐ Recommended by family/friends ☐ Other: _________

How severe do you consider your illness when you seek care? ☐ Mild ☐ Moderate ☐ Severe

How often do you visit a healthcare facility in a typical year? ☐ 0-1 times ☐ 2-3 times ☐ 4-6 times ☐ >6 times

Section 3: Factors Influencing Choice of Healthcare Provider

How important are the following factors when choosing a healthcare provider? Response scale: 1 = Not important 2 = Slightly important 3 = Moderately important 4 = Important 5 = Very important

Medical technology and equipment

Physician expertise

Hospital reputation

Waiting time

Distance from home

Cost of care

Insurance reimbursement

Service attitude of staff

Cleanliness and environment

Convenience of appointment scheduling

Recommendations from family/friends

Previous experience with the facility

Section 4: Satisfaction With Previous Healthcare Experiences

How satisfied were you with your most recent healthcare visit? Response scale: 1 = Very dissatisfied 2 = Dissatisfied 3 = Neutral 4 = Satisfied 5 = Very satisfied

Satisfaction with diagnosis

Satisfaction with treatment

Satisfaction with communication

Satisfaction with waiting time

Satisfaction with staff attitude

Overall satisfaction

Section 5: Future Healthcare Preferences

Which type of facility would you prefer for future care? ☐ Community health center ☐ Secondary hospital ☐ Tertiary hospital ☐ Private clinic

Main reason for this preference: ☐ Better doctors ☐ Better equipment ☐ Shorter waiting time ☐ Lower cost ☐ Insurance coverage ☐ Closer to home ☐ Better past experience ☐ Other: _________

Would you consider using a community health center if service quality improved? ☐ Yes ☐ No ☐ Not sure

Would you switch to a lower‑level facility if waiting times were shorter? ☐ Yes ☐ No ☐ Not sure
